# Self-stigma in alcohol dependence scale: development and validity of the short form

**DOI:** 10.1186/s12888-024-06187-z

**Published:** 2024-10-25

**Authors:** Sophia Rieckhof, Anya Leonhard, Stephanie Schindler, Juliane Lüders, Nicole Tschentscher, Sven Speerforck, Patrick W. Corrigan, Georg Schomerus

**Affiliations:** 1 Department of Psychiatry and Psychotherapy, Medical Faculty, University of Leipzig, Leipzig, Germany; 2 Department of Psychiatry and Psychotherapy, Medical Center, University of Leipzig, Leipzig, Germany; 3 Specialist Hospital Bethanien Hochweitzschen, Clinic for addiction medicine, Hochweitzschen, Germany; 4 Department of Psychology, Illinois Institute of Technology, Chicago, IL USA

**Keywords:** Internalized stigma, Substance abuse, Addiction, Questionnaire validation

## Abstract

**Background:**

Self-stigma is associated with low self-esteem, high shame and reduced drinking-refusal self-efficacy in people with alcohol use disorder (AUD). The Self-Stigma in Alcohol-Dependence Scale-Short Form (SSAD-SF) was designed to enable a brief, but valid assessment of AUD self-stigma.

**Methods:**

We reduced the 64-item SSAD, originally derived from 16 stereotypes towards people with AUD, by removing the most offensive items based on perspectives of people with lived experience. The newly created scale was then assessed and validated in a cross-sectional study involving 156 people reporting alcohol issues in various treatment settings.

**Results:**

The 20-item SSAD-SF includes five stereotypes, with good internal consistency for each subscale and the overall scale. It reflects the four-stage progressive model of self-stigmatization with decreasing scores over the stages *awareness of stereotypes*,* agreement with stereotypes*,* self-application of stereotypes*, and *harmful consequences for self-esteem*, and highest correlations between adjacent stages. The subscales *apply* and *harm* were associated with internalized stigma, shame, reduced self-esteem, and lower drinking-refusal self-efficacy, as supported by multivariate regression models.

**Discussion:**

The SSAD-SF is a valid instrument for measuring the process of self-stigmatization in people with AUD. Self-stigma is a consistent predictor of reduced self-esteem, higher shame and lower drinking-refusal self-efficacy in people with AUD. We discuss merits of the progressive model for understanding and addressing self-stigma in AUD.

**Supplementary Information:**

The online version contains supplementary material available at 10.1186/s12888-024-06187-z.

## Introduction

Alcohol use disorder (AUD) is common. According to the World Health Organization (WHO), 237 million men and 46 million women, or 1.4% of the global population, fulfil diagnostic criteria for AUD [1]. Furthermore, the WHO forecasts an increase of the global total alcohol per capita consumption in the next years [1]. High alcohol use contributes significantly to the global burden of disease and injury. In 2016, harmful alcohol use resulted in 3 million deaths worldwide, surpassing tuberculosis, HIV/AIDS, and diabetes in mortality [1]. Even though many people worldwide are affected, help-seeking is often delayed for many years [2], partially to avoid stigma and discrimination [3–5]. Compared to other mental disorders, people with AUD are less frequently seen as having a mental illness, are held more responsible for their illness, face increased social rejection and negative emotions, and are particularly impacted by structural discrimination, as highlighted in systematic reviews [6, 7]. At the societal level, surveys of population-representative samples of German adults consistently show that alcohol use disorder (AUD) receives low priority in healthcare funding [8]. While the consumption of most other addictive substances such as heroin, cocaine, cannabis or synthetic drugs is illegal and punished as criminal behavior, in most countries it is legal to drink alcohol, and drinking is even closely related to high-status events (e.g. fine dining, exclusive social events, or cultural and sporting events), despite its harmful consequences. This peculiarity creates discrepancy between generally accepted and even desirable ´cultivated drinking´ in the middle of society on the one hand and harmful consumption associated with stigma, marginalization and stereotypes on the other (e.g. the person is to blame for their alcohol consumption disorder, is weak-willed, etc.). According to Link and Phelan [9], stigma is a process in which individuals are initially labeled and assigned to an outgroup. Subsequently, stereotypes and prejudices are attributed to them, leading to experiences of discrimination and increased social distance. *Public stigma* arises when the general population agree with stereotypes and chooses to discriminate against individuals identified as having a mental illness [10]. Public stigma can have far-reaching and complex consequences for persons with substance use disorders (SUD). Room [11] argues that stigmatization and the associated marginalization reinforce the link between poverty and the severity of SUD. For those affected, especially those with a lower socio-economic status and fewer social resources, this results in a social downward spiral and an increase in social inequality [11]. In addition, there is evidence that groups with low power and status in particular have a reduced capacity for self-regulation, which in turn can have a negative impact on substance use [12]. Discrimination as a chronic stressor and the delayed help-seeking behaviour of people with SUD have negative impacts on their mental and physical health [13–15]. Moreover, public stigma can shape personal attitudes of persons, who are affected of SUD, and can lead to an internalization of negative stereotypes termed *self-stigma* [16] which is closely linked to feelings of shame, embarrassment, guilt [17] and reduced self-esteem [18]. Nonverbal displays of shame in people with a history of AUD are a predictor for future relapse and general deterioration of health [19]. Consistent with these findings, self-stigma is associated with lower drinking-refusal self-efficacy [20]. Awareness of personal failures, including loss of self-control and the inability to live according to one´s personal standards for a good life [21], may lead to self-identification as an ´addict´, increasing shame and damaging self-esteem [22, 23]. This process results in diminished self-respect and the ´why try´ effect [24], in which individuals doubt their self-worth and subsequently neglect or abandon personal values and life goals (e.g., job seeking, making friends, or reducing alcohol consumption). Given that alcohol consumption is common and generally accepted in society, it often falls to the individual to recognize and acknowledge any issues with their drinking habits [6, 25, 26]. The threat of self-stigmatization could make the painful admission that one’s own alcohol consumption is problematic even more difficult. By trivializing their alcohol consumption and thus avoiding acknowledging that they have an alcohol problem, individuals with AUDs avoid the label as an ‘alcoholic other’ and the associated self-destructive consequences of self-stigmatization [27, 28].

Conceptualizing the interrelation of public stigma and self-stigma, Corrigan [29] developed the progressive model of self-stigma of mental illness, which stages are measured by the Self-Stigma of Mental Illness Scale (SSMI; [30]). The model illustrates a ´trickle-down´ process of internalizing public stigma, beginning with awareness of negative stereotypes (1. *aware*) and personal agreement (2. *agree*). Developing a mental health problem can lead to self-application of these stereotypes (3. *apply*), resulting in lower self-esteem (4. *harm*). Schomerus et al. adapted the SSMI for people with AUD, creating the Self-Stigma in Alcohol Dependence Scale (SSAD; [20], which has excellent reliability and validity, consistent with Corrigan’s four-stage self-stigma model. Milan and Varescon [31] also confirmed the progressive model through correlations and repeated-measures analysis of variance in a French sample of individuals with AUD. However, the SSAD is a comprehensive instrument with a total of 64 items, limiting its value in routine clinical use and in studies measuring several distinct constructs. While the length of the scale might pose difficulties for respondents with impaired concentration, some of the negative stereotypes referred to in the scale might also be perceived as offensive by respondents, thus impairing readiness to respond and further intensifying the process of self-stigmatization and its consequences.

The aims of this work are to use two different samples to (1) develop an SSAD short form (SSAD-SF); (2) confirm its ability to replicate Corrigan’s self-stigmatization model; (3) evaluate its psychometric properties, including convergent validity with social distance (expected associations with subscale *agree*), internalized stigma (expected associations with subscales *agree*,* apply*,* harm*), and self-stigma-related constructs: self-esteem, shame, and drinking-refusal self-efficacy; and (4) to examine whether self-stigma (represented by subscales *apply*,* harm*) can predict self-esteem, shame, and drinking-refusal self-efficacy, even when controlling for depressive symptoms and gender, thus establishing criterion validity.

## Methods

### Development of the self-stigma in alcohol dependence scale short form

We adopted the SSAD [20] consisting of four subscales (*aware*,* agree*,* apply*,* harm*), similar to the SSMI [30]. Each subscale consists of the same 16 negative stereotypes, presented in different order, preceded by 4 different introductory phrases. Stereotype awareness (*aware*) is introduced with ´I think the public believes most persons with alcohol problems are´, after which respondents rate each stereotype accordingly. The subscale for stereotype agreement (*agree*) begins with ´I believe most persons with alcohol problems are´, stereotype application (*apply)* after ´because I have alcohol problems, I am´, and self-esteem decrement (*harm*) with ´I currently respect myself less because I am´ All items are answered with a 5-point Likert scale from 1 ´I strongly disagree´ to 5 ´I strongly agree´. The SSAD shows excellent reliability (Cronbach´s α = 0.86–0.93) and validity in measuring the four stages of self-stigmatization [20]. All stereotypes performed equally well, leaving no obvious indication for which items to leave out. Therefore, we chose a *two-step process* to reduce the number of items. In the first step, drawing from a method used by Corrigan et al. [32], we excluded items that participants of self-help groups with current or previous AUD (*n* = 12) rated as being most offensive on a 5-point Likert scale (the higher, the more offensive). Participants were recruited directly through an out-patient addiction counseling center in February 2020 in Saxony, Germany. All participants filled out the paper-and-pencil questionnaire on-site and returned it along with a signed informed consent agreement. In the second step, we analyzed item qualities (i.e. item analysis, reliability) for the remaining items in a sample of persons with AUD (*n* = 156; description see 2.2).

### Reliability and validity of the SSAD-SF

To assess internal consistency, we used the SSAD validation dataset [20] comparing the mean item-total correlations of the selected and remaining items. To further evaluate reliability, we compared the SSAD-SF’s performance in replicating Corrigan’s self-stigmatization model, its psychometric properties, and its ability to predict the negative consequences of self-stigma with the data from the long-version validation survey [20]. This comparison provides insights into the replicability of results across different samples of individuals with alcohol problems, and thus into the reliability of both the SSAD and its short form.

***Participants and procedures.*** We surveyed people who undergoing in-patient detoxification and 2–3 weeks of rehabilitation at a psychiatric substance use treatment facility, rehabilitation treatment, or who were affiliated with an out-patient addiction counseling center or self-help group between March 2021 and June 2022 in Saxony, Germany. We did not establish formal diagnoses, instead, we included patients who self-identified as having an alcohol-related problem and were at least 18 years old. Either recruiters or treatment providers handed 260 paper-and-pencil questionnaires out on-site. Of these, 156 (60%) were returned along with a signed agreement of informed consent. The study was approved by the responsible Ethics Committee of the University of Leipzig (169/201-ek).

***Polysubstance use*** was measured with the item: ´In addition to your problematic alcohol consumption, is there problematic consumption of medication or illegal drugs (e.g. cannabis, MDMA, heroin, etc.)?´. Answer categories were ´no´ and ´yes´ with the subcategory ´if yes, which?´.

***Comorbidity*** was measured with the items ´have you been diagnosed with another mental illness?´ and ´have you been diagnosed with a serious physical illness?´. Answer categories were ´no´ and ´yes´ with the subcategory ´if yes, which one?´.

***Instruments.*** We used the following measures to evaluate the convergent validity of the SSAD-SF to elicit constructs related to the self-stigmatization process.

***Social distance***. The Social Distance Scale (SDS) published by Link et al. [33] measures the desire to avoid contact with a particular group of people and is an established instrument for measuring discriminatory attitudes towards people with mental illness [34]. We used the adapted version of Angermeyer and Matschinger [35], which asks whether respondents are willing to engage in several forms of every day contact with ´someone having an alcohol problem´. It consists of seven items. Responses were recorded with a 5-point Likert scale (´definitely not´ to ´definitely´), resulting in a sum score of 7 to 35 points. For our analysis, scores were reversed, so higher values signify a greater desire for social distance. Internal consistency was high (Cronbach´s α = 0.82).

***Internalized Stigma***. To measure self-stigma of people with mental illness, we used the established Internalized Stigma of Mental Illness Scale (ISMI; [36]). Several studies have applied the ISMI to people with SUD in different countries (e.g [37, 38]). The internal consistency reliability of the used short form [39] is adequate (Cronbach´s α = 0.75). We adapted the German translation by Sibitz et al. [40] by replacing the term ´mental illness´ with ´addiction disorder´. Answers were given on 4-point Likert scales (´strongly disagree´ to ´strongly agree´). The mean of the total score was used. Internal consistency was adequate (Cronbach´s α = 0.79).

To assess the criterion validity of self-stigma assessed by the SSAD-SF, we examined the extent to which self-stigma predicts negative effects on self-esteem, shame, and drinking-refusal self-efficacy while controlling for depressive symptoms and gender.

***Self-esteem***. The Rosenberg Self-Esteem Scale (RSES) published by Rosenberg [41] is a valid and frequently used measure [42] consisting of 10 items that are answered using a 4-point Likert scale (´strongly disagree´ to ´strongly agree´). High sum scores (range 0–30) indicate stronger self-esteem, while values below 15 indicate low self-esteem. Our data showed high internal consistency (Cronbach´s α = 0.83).

***Shame***. We assessed shame with the following item: ´I feel ashamed because I have an alcohol problem´. On a 5-point Likert scale, participants rated *perceived frequency* and *experienced intensity*. We used the mean score of both items. Internal consistency was high (Spearman-Brown-coefficient = 0.92).

***Drinking-refusal self-efficacy***. The ´Kurzfragebogen zur Abstinenzzuversicht´ [“Short Questionnaire on Abstinence Confidence”, KAZ-35] by Körkel and Schindler [43] is a validated and based on the Situational Confidence Questionnaire [44]. Participants are asked to rate their confidence in being able to resist drinking in 35 abstinence-threatening situations on a 10-point scale (´not confident at all´ to ´totally confident´). The mean score was used and the internal consistency was high (Cronbach’s alpha = 0.97).

***Depressive Symptoms***. We used the ´Patient Health Questionnaire eight-item depression scale´ (PHQ-8), which has been established as a valid assessment of depression severity [45]. Respondents are asked to rate the frequency of eight core symptoms of depression as experienced within the past two weeks on a 4-point Likert scale (´not at all,´ to ´nearly every day´). Sum scores vary from 0 to 24, indicating mild (5–9), moderate (10–14) or severe depressive symptoms (15–24). In our sample, 31.5% showed mild, 20.8% moderate and 31.5% severe depressive symptoms with high internal consistency (Cronbach´s α = 0.90).

### Statistical analyses

For item reduction of the SSAD long form we calculated the means of the offensiveness-rating and set a cut off value which was oriented on the overall mean value of the offensiveness-score. Item-total correlations were calculated to compare selected with remaining items by using the dataset of the validation of the long form of the SSAD [20].

For an extended reliability test, we also carried out the following calculations on the dataset used for the validation of the long version of the SSAD in 2010. This enabled us to compare between the different samples from 2010 to 2022. First, we calculated item distributions, item difficulty, and item discriminatory power for all SSAD-SF items and tested for goodness of fit for a psychometric characterization of the new measure. Second, we calculated Cronbach’s alpha as an indicator of the reliability of the SSAD-SF subscales and total score. We then tested the validity of the measure in several ways. In order to test the progressive character of the subscales (i.e. the sequential nature of the stages, with sum scores decreasing from *aware* to *harm*, and proximate stages being most closely correlated), we used a repeated measures ANOVA with Mauchly test for sphericity, according to guidelines of Girden [46], Greenhouse-Geisser correction for 2010 data and Huynh-Feldt correction for 2022 data was used. We applied a contrast analysis for proximate SSAD-SF scales with Bonferroni-Holm correction. A two-tailed alpha of 0.05 was used. We established convergent validity by calculating bivariate correlations among SSAD-SF subscales, and between SSAD-SF subscales and social distance (SDS), internalized stigma (ISMI), self-esteem (SES), shame, drinking-refusal self-efficacy (KAZ-35), and depressive symptoms (PHQ-8). The requirements for Pearson product moment correlations were met.

Finally, we analyzed the relationship between the final stages of the self-stigmatization process (subscales *apply* and *harm*) as predictor variables with self-esteem, shame, and drinking-refusal self-efficacy as criterion variables in multiple linear regression analyses. We included depressive symptoms and gender as control variables to assess whether the relationship between self-stigma and predictor variables remained significant after accounting for their shared variance. For each outcome, Model 1 was calculated with depressive symptoms and gender as criterion variables only, while Models 2 and 3 incorporated either *apply* or *harm*, respectively, as additional predictors. Due to an error in data collection, there was 10% missing data (*n* = 16) for age. For this reason, we decided to omit age as a covariate from multiple regression calculations. Linearity between predictors and criteria was demonstrated using partial regression plots. Normal distributions of the residuals of the predictor variables were verified using histograms, P-P plots, and Kolmogorov-Smirnov tests. Outliers were controlled by case-by-case diagnostics, studentized residuals, leverage values, and Cook distances. We excluded an extreme outlier from our regression analyses. All statistical analyses were conducted using SPSS 27 (IBM SPSS Statistics for Windows, version 27).

## Results

### Demographic characteristics

Our first sample consisted of 7 men (58%) and 5 women (42%) with an average age of 59.1 years (SD = 8.5). The second sample included 94 (60%) male and 62 female (40%) participants and with mean age of 48.4 years (SD = 11.6), 74 (48%) lived in a stable relationship and 78 (50%) were employed. For detailed sample characteristics see Table 1. For details on the sample of the SSAD validation study, see Schomerus et al. [20].

**Table 1:** Sample characteristics, except for n all data are presented in percent

n		Total sample	In-patient detoxification	Rehabilitation treatment	Outpatient setting
		156	90 (57.7%)	35 (22.4%)	31 (19.9%)
Data in %					
Gender	Male	60.3	54.4	68.6	67.7
	Female	39.7	45.6	31.4	32.3
Age	21–35	11.9	10.3	22.2	6.7
	36–50	43.7	53.8	44.4	16.7
	51–65	39.3	34.6	29.6	60.0
	≥ 66	5.2	1.3	3.7	16.7
Educational level	High	25.7	34.5	17.2	9.7
	Middle	51.9	48.9	45.7	67.7
	Low	17.3	13.3	22.9	22.6
	No graduation	5.1	3.3	14.3	-
Marital status	Single	30.1	29.2	50.0	12.9
	Partnership/Married	48.1	51.8	23.5	61.3
	Divorced/widowed	21.2	18.0	26.5	25.8
Housing situation	Living alone	41.6	40.0	51.5	35.5
	Cohabitating	51.3	57.8	21.2	64.5
	Homeless	7.1	2.2	27.3	-
Employment status	Employed^1^	50.0	57.8	37.1	41.9
	Unemployed	28.8	26.7	45.7	16.1
	Disability pension/ Reduced earning Capacity pension	8.3	7.8	8.6	9.7
	Retirement pension	5.8	1.1	2.9	19.4
	Other	7.1	5.6	5.7	12.9
Current abstinence period over 6 months		21.8	3.3^2^	25.7	71.0
Polysubstance use		25.0	16.7	54.3	16.1
Comorbidity	Other mental illness	42.3	47.8	31.4	38.7
	Somatic illness	22.4	18.9	25.7	29.0

^1^including participants on sick leave

^2^In order to prevent an imminent relapse, those affected who are abstinent can also take advantage of qualified withdrawal therapy if there are signs of imminent destabilization

### Item reduction of the SSAD

The overall mean value of the offensiveness rating in the first sample was M = 3.3, and a cut-off value of 3.3 was applied based on this, which excluded 10 of 16 stereotypes. In the second sample, the stereotype ´(…) are to blame for their problems´ performed poorly with a corrected discriminatory power of < 0.3 and low internal consistencies at subscale level and was therefore excluded.

The final questionnaire includes 4 sets of 5 stereotypes, 20 items in total (for the questionnaire see supplement: Additional file 2). Sum scores for the four subscales ranged from 5 to 25.

### Reliability and validity of the SSAD-SF

When comparing the means of item-total correlations of the 5 selected and the 11 remaining stereotypes, we found no significant differences (see supplement Tables S1, S2). Results of item distributions and item difficulties for all SSAD-SF items can also be found in the supplement (Table S3). Mean scores, standard deviations, and internal consistencies for the 4 × 5 selected SSAD-SF items from 2010 to 2022 are shown in the upper half of Table 2. Except for a higher Cronbach’s alpha in the SSAD-SF subscale *harm* in 2022, the two datasets were largely indistinguishable. Based on 2022 data, corrected item-subscale correlations ranged from 0.19 to 0.62 (see supplement Table S3) and can thus be interpreted as adequate. The SSAD-SF total scale showed good internal consistency with Cronbach´s Alpha of 0.84. The bottom half of Table 2 lists stigma related constructs, which showed good to excellent reliability coefficients.

**Table 2:** Mean, standard deviation and internal consistencies of the SSAD-SF

	2010			2022		
	Mean	SD	Reliability coefficient^1^	Mean	SD	Reliability coefficient^1^
SSAD-SF aware	15.29	5.05	0.81	16.06	4.28	0.71
SSAD-SF agree	13.33	4.25	0.76	12.79	3.73	0.70
SSAD-SF apply	10.63	3.82	0.66	10.68	3.95	0.66
SSAD-SF harm	10.20	4.18	0.55	9.96	4.21	0.73
SSAD-SF total score	12.36	3.48	0.88	12.30	2.97	0.84
stigma related constructs						
SDS social distance	23.67	6.71	0.88	21.35	5.86	0.82
ISMI internalized stigma				2.29	0.55	0.79
RSES self-esteem				18.88	5.97	0.83
shame				3.05	1.23	0.92
KAZ-35 drinking-refusal self-efficacy	70.77	25.83	0.97	67.93	22.08	0.97
PHQ-8 depression				10.48	6.18	0.90

Values are based on data from 2010 (*N* = 120–153) and 2022 (*N* = 137–156)

^1^for shame the Spearman-Brown-coefficient was calculated, for all the other scales, Cronbach´s Alpha is reported

SSAD-SF: Self-Stigma in Alcohol Dependence Scale short form; aware: awareness of stereotypes; agree: agreement with stereotypes; apply: self-application of stereotypes; harm: harmful consequences for self-esteem

Consistent with the model of Corrigan and colleagues [29], means decreased from *agree* to *harm* as progressive stages of self-stigmatization in data from both 2010 and 2022. Repeated measures ANOVA determined a significant difference in mean scores between these four stages (2010: F(2.00, 285.52) = 98.92, *p* < 0.001, partial η²=0.41; 2022: F(2.54, 372.98) = 120.27, *p* < 0.001, partial η²=0.45). Subsequent repeated contrasts determined significant differences in mean scores (*p* < 0.001) between *aware* and *agree* (2010: F(1,143) = 50.28; 2022: F(1,147) = 90.62), and for *agree* and *apply* (2010: F(1;143) = 87.58; 2022: F(1,147) = 49.50). For *apply* and *harm*, differences did not reach significance in 2022 data (2010: F(1,143) = 4.06, *p* = 0.046; 2022: F(1,147) = 3.33, *p* = 0.070).

The correlations between the four stages revealed the highest values for proximate stages and the lowest for distant stages, as presented in the top half of Table 3. The construct validity of the SSAD-SF scales, as illustrated in the lower half of Table 3, is demonstrated through correlations with content-related scales. *Social distance* is associated with higher scores in *agree* and *harm*. Internalized stigma correlated with *agree*, *apply* and *harm* significantly. Associations between self-esteem, shame and drinking-refusal self-efficacy provided content validation of the self-stigma scales *apply* and *harm*. Low self-esteem, shame, and low drinking-refusal self-efficacy were all associated with higher scores in the subscales *apply* and *harm*, but not significantly with *aware* and *agree*.

**Table 3:** Pearson´s product moment correlations for the SSAD-SF subscales and related constructs

	SSAD-SF aware		SSAD-SF agree		SSAD-SF apply		SSAD-SF harm	
	2010	2022	2010	2022	2010	2022	2010	2022
SSAD-SF aware	1.00	1.00	0.73**	0.32**	0.39**	0.17*	0.31**	0.21*
SSAD-SF agree					0.55**	0.48**	0.49**	0.31**
SSAD-SF apply							0.71**	0.67**
SSAD-SF harm							1.00	1.00
stigma related constructs								
SDS social distance	0.22**	0.16	0.40**	0.29**	0.12	0.27**	0.20*	0.36**
ISMI internalized stigma		0.17*		0.41**		0.56**		0.46**
RSES self-esteem		− 0.10		− 0.16*		− 0.54**		− 0.54**
shame		− 0.01		− 0.02		0.32**		0.35**
KAZ-35 drinking-refusal self-efficacy	− 0.13	0.05	− 0.12	− 0.17*	− 0.37**	− 0.32**	− 0.34**	− 0.35**
PHQ-8 depression		0.04		0.08		0.40**		0.35**

SSAD-SF: Self-Stigma in Alcohol Dependence Scale short form; aware: awareness of stereotypes; agree: agreement with stereotypes; apply: self-application of stereotypes; harm: harmful consequences for self-esteem

* *p* < 0.05

** *p* < 0.01

Table 4 shows the results of multiple linear regression analyses examining a potential influence of *apply* and *harm* on self-esteem, shame, and drinking-refusal self-efficacy. Across all outcome variables, *apply* and *harm* were independent significant predictors for self-esteem, shame, and drinking-refusal self-efficacy, further increasing the amount of variance explained by depressive symptoms and gender by 3–4% (shame), 3–8% (drinking-refusal self-efficacy) and 15% (self-esteem). Women showed significantly higher shame. The negative association between depressive symptoms and drinking-refusal self-efficacy was no longer significant when introducing *apply* or *harm* into the model. We report results using mean imputed age scores as sensitivity analysis and results of a subgroup analysis of probands who currently self-reported abstinence (see supplement Tables S4, S5).

**Table 4:** Linear regression models

	Criterion variables																	
	Self-esteem (RSES)						Shame						Drinking-refusal self efficacy (KAZ-35)					
	Model 1		Model 2		Model 3		Model 1		Model 2		Model 3		Model 1		Model 2		Model 3	
N	148		147		147		154		147		147		150		144		144	
R²	0.223		0.373		0.374		0.259		0.293		0.301		0.068		0.153		0.098	
***Predictor variables***	**Beta**	**p**	**Beta**	**p**	**Beta**	**p**	**Beta**	**p**	**Beta**	**p**	**Beta**	**p**	**Beta**	**p**	**Beta**	**p**	**Beta**	**p**
Gender	0.01	0.900	0.01	0.857	0.05	0.454	− 0.22	0.003	− 0.23	0.002	− 0.25	< 0.001	0.07	0.377	0.08	0.337	− 0.09	0.296
Depression (PHQ-8)	− 0.47	< 0.001	− 0.30	< 0.001	− 0.30	< 0.001	0.44	< 0.001	0.38	< 0.001	0.37	< 0.001	− 0.24	0.003	− 0.12	0.182	− 0.17	0.600
SSAD-SF apply			− 0.42	< 0.001					0.16	0.040					− 0.32	< 0.001		
SSAD-SF harm					− 0.42	< 0.001					0.19	0.016					− 0.19	0.036

SSAD-SF: Self-Stigma in Alcohol Dependence Scale short form; self-application of stereotypes; harm: harmful consequences for self-esteem

## Discussion

The 20-item SSAD short form (SSAD-SF) proved to be a reliable and valid instrument measuring the progressive stages of AUD self-stigmatization, potentially facilitating the assessment of self-stigma in people with AUD with fewer, and less offensive items.

Before discussing the implications of our study, we discuss its methodological strengths and weaknesses. We consider the participation of people with lived experience of AUD and their perspectives on perceived stereotype offensiveness in the first phase of item reduction a major strength in the development of the SSAD-SF. Nevertheless, possible biases in the *two-step process* of item reduction cannot be ruled out. The focus group consisted exclusively of people who were active in a self-help group. It is conceivable that the inclusion of those affected from other contexts would have provided a more comprehensive picture of this target group. Furthermore, the determination of the cut-off value is debatable: we used the mean value of the perceived stereotype offensiveness as a reference, but there are also alternative methods for determining a cut-off value for item reduction (e.g. use of the median, determination of the cut-off value on the basis of expert opinions or practical considerations). In the second step of item reduction, the low discriminatory power and internal consistencies indicated that the item ´(…) are to blame for their problems´ differed from the underlying construct of the other included stereotypes, which is why we decided to exclude this stereotype. Although blame is a core aspect that distinguishes the public stigma of substance use disorders from other mental disorders like depression or schizophrenia [7], we decided to omit it from our self-stigma scale to improve its internal consistency.

Compared to the original 64-item version, the SSAD-SF showed somewhat lower reliability in terms of internal consistency. However, the brevity and accessibility of the SSAD-SF, resulting potentially higher response rates, and more diverse samples must be weighed against a higher test quality. Due to the selection of stereotypes from less offensive items, some facets of AUD stigma may no longer be fully represented. Although we have worked with the short version to stigmatize as little as possible through the items, a potential stigmatization of the respondents cannot be excluded. This makes it all the more important to inform the participants about the self-stigmatization process and to give them the opportunity to discuss any questions or feelings that arise after completing the SSAD scale. Compared to the initial study with the SSAD, we included a broader spectrum of people with AUD (e.g. more women, people from out-patient settings and with stable abstinence). However, our sample only involved people seeking some form of help. Further studies are needed that include individuals who are not (yet) seeking help. Finally, it should be emphasized that our cross-sectional, observational study can only test correlations, but not draw any conclusions about time courses and causality between the self-stigma scales, self-esteem, shame and drinking-refusal self-efficacy.

Our study additionally confirmed Corrigan’s four-step progressive model of self-stigmatization [20]. Depicting this entire process in one scale distinguish the SSMI, SSAD and SSAD-SF from other measures of self-stigma (e.g. ISMI [36], ; SASSS [47], ; SU-SMS [48]). The advantage of these scales is that they make it possible to measure public stigma towards people with AUD and the subsequent self-stigmatization as processes that build on each other. This enables a better understanding of the relationships between them. The use of the SSAD short form enables individuals with AUD who are in treatment, counselling or self-help to better understand the process of self-stigmatization and its consequences based on their own experiences when answering the items. It allows them to localize themselves on these steps and stimulates an exchange on this basis, both in individual contact and in group settings. Ideally, the scale can help clarify the profound consequences of public and internalized stigma, question one’s own internalized stereotypes, strengthen or establish personal resources, and develop individual stigma resistance. Moreover, the short form of the SSAD has the advantage over the long version as it does not overburden participants in this setting due to its brevity. For research purposes, the *aware* and *agree* scales can be used in healthy populations and enable comparisons across affected and unaffected groups. Most importantly, linking self-stigmatization to public with the progressive model makes it possible to draw differentiated conclusions and develop novel hypotheses about the origin and maintenance of self-stigma in specific groups. In a study of people with AUD and their self-stigmatization related to childhood trauma, Stolzenburg et al. [49] found that experiencing childhood trauma was linked to more severe self-stigma. The difference between people with and without childhood trauma in terms of self-stigma was not restricted to stronger *apply* and *harm* agreement, but originated in stronger personal *agreement* with negative stereotypes about other people with AUD. They hypothesized that past negative experiences with other people who use alcohol, such as parents, contributed to childhood trauma and to an especially negative view of others with AUD. This, in turn, made one’s own alcohol problem especially damaging to self-esteem. Employing the SSAD short form could economically test whether the results of Stolzenburg and colleagues can be replicated. In the next step, the scale can be utilized to explore the hypothesis that stressful childhood experiences, when combined with problematic alcohol consumption in the immediate family, lead to a more pronounced rejection of individuals with AUD and to self-rejection, as those affected fear following in the footsteps of their parents. If confirmed, these findings suggest the need for interventions focused on addressing trauma and life experiences to reduce self-stigma in this group.

Our results showed good construct validity of the SSAD short form through moderate to strong correlations of self-stigma with social distance, internalized stigma, self-esteem, shame and drinking-refusal self-efficacy. Our findings indicate that women who self-identify as having an alcohol problem experience higher levels of shame compared to men. This aligns with the observations of O’Connor and Berry et al. [44], who investigated individuals with SUD in recovery. Shame can result from self-stigma, but gender differences in self-stigma among individuals with SUD have not yet been adequately researched. Initial studies on self-stigma in individuals with SUD [50] or schizophrenia [51, 52] suggest heightened self-stigma in women. Additionally, Else-Quest et al. [53] demonstrated in their meta-analysis that women generally experience slightly higher levels of shame than men, which could influence our results independently of self-stigmatization. Nonetheless, it remains crucial to consider potential differences in self-stigmatization among men, women, and non-binary individuals [54] in SUD. This consideration is essential, as SUD stereotypes may be gender-specific, causing individuals of different genders to internalize distinct stereotypes to a greater extent. This, in turn, can have implications for awareness of the problem, motivation for treatment, and provide specific points of focus for interventions. Furthermore, existing literature highlights broader gender-based distinctions in the development, progression, health implications, psychosocial consequences, and treatment outcomes of SUD [55–58]. These findings underscore the importance of adopting a gender-sensitive approach in researching individuals with SUDs, aiming to promote a more equitable approach to prevention, treatment, and anti-stigma campaigns.

Finally, our results showed that self-stigma predicted lower self-esteem, increased shame, and lower drinking self-efficacy, even when controlling for depressive symptoms and gender. This suggests good criterion validity. Compared to the validation study [20] it is noticeable that the association between *harm* and *drinking-refusal self-efficacy* in the linear regression model was somewhat lower. One possible explanation could be the more diverse sample examined in the present study, which included people practicing abstinence. Our subgroup analysis revealed that self-stigma was not or low associated with shame and drinking-refusal self-efficacy for probands with abstinence, in contrast to non-abstinent participants. Due to the small sample size of the subgroup analysis, no conclusions can be drawn for people living in abstinence. But based on our results, trends that need to be replicated in future studies, including larger sample sizes, suggest that a longer period of abstinence could serve as a protective factor against the negative consequences of self-stigma and promote stigma resilience, highlighting it as a worthwhile topic for further research.

In summary, our study supports the findings that higher self-stigma in people with AUD is associated with lower self-esteem, more shame, and less drinking-refusal self-efficacy. Self-stigma could thus prove a worthwhile target for interventions in people with AUD. The SSAD-SF showed good test performance and offers a brief, economic way to measure the process of self-stigmatization in people with AUD.

## Electronic supplementary material

Below is the link to the electronic supplementary material.

Supplementary Material 1

Supplementary Material 2

## Data Availability

The data underlying this article cannot be shared publicly due to the privacy of individuals that participated in the study. The data will be shared on reasonable request to the corresponding author.

## Abbreviations

AUD: Alcohol use disorder
ISMI: Internalized Stigma of Mental Illness Scale
PHQ: Patient Health Questionnaire depression scale
KAZ: Short Questionnaire on Abstinence Confidence
SSAD: Self-Stigma in Alcohol-Dependence Scale
SSAD-SF: Self-Stigma in Alcohol-Dependence Scale-Short Form
SDS: Social Distance Scale
SUD: Substance use disorder
RSES: Rosenberg Self-Esteem Scale
