# Supplementary material for: Self-stigma in alcohol dependence scale: development and validity of the short form
Source: BMC Psychiatry. 2024 Oct 25;24:735. doi: 10.1186/s12888-024-06187-z (PMC11515145; doi:10.1186/s12888-024-06187-z)
Supplement: Supplementary file 1 — Supplementary Material 1 [file 12888_2024_6187_MOESM1_ESM.docx]

## ***Supplement: Additional file 1***

**Table S1** Corrected item-total correlation from the 2010 data of the 5 selected stereotypes

|  | Subscale | Item | Corrected item-total correlation | Means of corrected  item-total correlation |
| --- | --- | --- | --- | --- |
|  | *aware* | *I think the public believes…* |  |  |
| 1 |  | most people with alcohol problems are unable to get or keep a regular job. | .43 | .54 |
| 2 |  | most people with alcohol problems are unpredictable. | .57 |  |
| 3 |  | most people with alcohol problems are emotionally unstable. | .61 |  |
| 4 |  | most people with alcohol problems will never get away from alcohol. | .49 |  |
| 5 |  | most people with alcohol problems are self-pitying. | .63 |  |
|  | *agree* | *I think…* |  |  |
| 6 |  | most people with alcohol problems are unpredictable. | .61 | .48 |
| 7 |  | most people with alcohol problems will never get away from alcohol. | .51 |  |
| 8 |  | most people with alcohol problems are unable to get or keep a regular job. | .51 |  |
| 9 |  | most people with alcohol problems are self-pitying. | .65 |  |
| 10 |  | most people with alcohol problems are emotionally unstable. | .62 |  |
|  | *apply* | *Because I have alcohol problems…* |  |  |
| 11 |  | I am emotionally unstable. | .52 | .46 |
| 12 |  | I am unable to get or keep a regular job. | .43 |  |
| 13 |  | I am self-pitying. | .53 |  |
| 14 |  | I will never get away from alcohol. | .48 |  |
| 15 |  | I am unpredictable. | .35 |  |
|  | *harm* | *I currently respect myself less, because…* |  |  |
| 16 |  | I am self-pitying. | .48 | .40 |
| 17 |  | I am unable to get or keep a regular job. | .43 |  |
| 18 |  | I am emotionally unstable. | .55 |  |
| 19 |  | I will never get away from alcohol. | .25 |  |
| 20 |  | I am unpredictable. | .30 |  |
|  |  |  |  | Total mean of corrected  item-total correlations= .48 |

aware: awareness of stereotypes; agree: agreement with stereotypes; apply: self-application of stereotypes; harm: harmful consequences for self-esteem

**Table S2** Corrected item-total correlation from the 2010 data of the 11 remaining stereotypes

|  | Subscale | Item | Corrected item-total correlation | Means of corrected  item-total correlation |
| --- | --- | --- | --- | --- |
|  | *aware* | *I think the public believes…* |  |  |
| 1 |  | most people with alcohol problems cannot be trusted | .36 | .52 |
| 2 |  | most people with alcohol problems are disgusting | .46 |  |
| 3 |  | most people with alcohol problems are dirty and unkempt. | .48 |  |
| 4 |  | most people with alcohol problems are to blame for their problems. | .34 |  |
| 5 |  | most people with alcohol problems are unreliable | .60 |  |
| 6 |  | most people with alcohol problems are below average in intelligence. | .49 |  |
| 7 |  | most people with alcohol problems are weak-willed. | .58 |  |
| 8 |  | most people with alcohol problems are violent. | .65 |  |
| 9 |  | most people with alcohol problems live on other people’s expenses. | .56 |  |
| 10 |  | most people with alcohol problems are unable to resolve conflicts other than with alcohol. | .59 |  |
| 11 |  | most people with alcohol problems are lazy. | .65 |  |
|  | *agree* | *I think…* |  |  |
| 12 |  | most people with alcohol problems are to blame for their problems. | .48 | .62 |
| 13 |  | most people with alcohol problems are dirty and unkempt. | .61 |  |
| 14 |  | most people with alcohol problems are lazy. | .64 |  |
| 15 |  | most people with alcohol problems are unable to resolve conflicts other than with alcohol. | .58 |  |
| 16 |  | most people with alcohol problems live on other people’s expenses. | .68 |  |
| 17 |  | most people with alcohol problems are violent. | .62 |  |
| 18 |  | most people with alcohol problems cannot be trusted. | .66 |  |
| 19 |  | most people with alcohol problems are weak-willed. | .60 |  |
| 20 |  | most people with alcohol problems are disgusting. | .61 |  |
| 21 |  | most people with alcohol problems are unreliable. | .72 |  |
| 22 |  | most people with alcohol problems are below average in intelligence. | .58 |  |

aware: awareness of stereotypes; agree: agreement with stereotypes; apply: self-application of stereotypes; harm: harmful consequences for self-esteem
**Note.** Table S2 continued on the next page

**Table S2** Corrected item-total correlation from the 2010 data of the 11 remaining stereotypes (continued)

|  | Subscale | Item | Corrected item-total correlation | Means of corrected  item-total correlation |
| --- | --- | --- | --- | --- |
|  | *apply* | *Because I have alcohol problems…* |  |  |
| 23 |  | I am unreliable. | .49 | .41 |
| 24 |  | I am lazy. | .45 |  |
| 25 |  | I am below average in intelligence. | .45 |  |
| 26 |  | I cannot be trusted. | .55 |  |
| 27 |  | I am weak-willed. | .44 |  |
| 28 |  | I am living on other people’s expenses. | .42 |  |
| 29 |  | I am violent. | .31 |  |
| 30 |  | I am dirty and unkempt. | .29 |  |
| 31 |  | I am unable to resolve Problems other than with alcohol. | .40 |  |
| 32 |  | I am to blame for my problems. | .36 |  |
| 33 |  | I am disgusting. | .32 |  |
|  | *harm* | *I currently respect myself less, because…* |  |  |
| 34 |  | I am lazy. | .32 | .33 |
| 35 |  | I am unreliable. | .39 |  |
| 36 |  | I am weak-willed. | .08 |  |
| 37 |  | I cannot be trusted. | .40 |  |
| 38 |  | I am to blame for my problems. | .36 |  |
| 39 |  | I am violent. | .28 |  |
| 40 |  | I am disgusting. | .31 |  |
| 41 |  | I am unable to resolve problems other than with alcohol. | .47 |  |
| 42 |  | I am dirty and unkempt. | .25 |  |
| 43 |  | I am living on other people’s expenses. | .45 |  |
| 44 |  | I am below average in intelligence. | .34 |  |
|  |  |  |  | Total mean of corrected  item-total correlations= .47 |

**Table S3** Item characteristics of the final 20 SSAD-SF-items

|  | Sub-scale | Item | Mean | Median | SD | Skewness | Kurtosis | Item difficulty | Corrected  item-subscale-  correlation |
| --- | --- | --- | --- | --- | --- | --- | --- | --- | --- |
|  | *aware* | *I think the public believes…* |  |  |  |  |  |  |  |
| 1 |  | most people with alcohol problems are unable to get or keep a regular job. | 3.43 | 3.0 | 1.14 | -.36 | -.56 | .53 | .21 |
| 2 |  | most people with alcohol problems are unpredictable. | 3.01 | 3.0 | 1.14 | -.07 | -.61 | .42 | .35 |
| 3 |  | most people with alcohol problems are emotionally unstable. | 3.62 | 4.0 | 1.04 | -.26 | -.51 | .57 | .46 |
| 4 |  | most people with alcohol problems will never get away from alcohol. | 3.23 | 4.0 | 1.33 | -.37 | -1.04 | .48 | .19 |
| 5 |  | most people with alcohol problems are self-pitying. | 3.10 | 3.0 | 1.16 | -.12 | -.61 | .45 | .39 |
|  | *agree* | *I think…* |  |  |  |  |  |  |  |
| 6 |  | most people with alcohol problems are unpredictable. | 2.50 | 2.0 | 1.07 | .36 | -.32 | .31 | .49 |
| 7 |  | most people with alcohol problems will never get away from alcohol. | 2.53 | 3.0 | 1.11 | .16 | -.82 | .31 | .28 |
| 8 |  | most people with alcohol problems are unable to get or keep a regular job. | 2.41 | 2.0 | 1.24 | .59 | -.61 | .30 | .44 |
| 9 |  | most people with alcohol problems are self-pitying. | 2.48 | 2.0 | 1.12 | .38 | -.58 | .31 | .45 |
| 10 |  | most people with alcohol problems are emotionally unstable. | 2.97 | 3.0 | 1.08 | .05 | -.61 | .41 | .46 |
|  | *apply* | *Because I have alcohol problems…* |  |  |  |  |  |  |  |
| 11 |  | I am emotionally unstable. | 2.71 | 3.0 | 1.34 | .30 | -1.07 | .37 | .52 |
| 12 |  | I am unable to get or keep a regular job. | 2.04 | 1.0 | 1.35 | 1.05 | -.22 | .23 | .39 |
| 13 |  | I am self-pitying. | 2.32 | 2.0 | 1.25 | .65 | -.57 | .28 | .62 |
| 14 |  | I will never get away from alcohol. | 1.88 | 2.0 | 1.05 | 1.18 | .86 | .18 | .34 |
| 15 |  | I am unpredictable. | 1.78 | 1.0 | 1.12 | 1.25 | .51 | .16 | .40 |

SSAD-SF: Self-Stigma in Alcohol Dependence Scale- short form, SD= standard deviation; aware: awareness of stereotypes; agree: agreement with stereotypes; apply: self-application of stereotypes; harm: harmful consequences for self-esteem
**Note.** Table S3 continued on the next page

**Table S3** Item characteristics of the final 20 SSAD-SF-items (continued)

|  | Sub-scale | Item | Mean | Median | SD | Skewness | Kurtosis | Item difficulty | Corrected  item-subscale-  correlation |
| --- | --- | --- | --- | --- | --- | --- | --- | --- | --- |
|  | *harm* | *I currently respect myself less, because…* |  |  |  |  |  |  |  |
| 16 |  | I am self-pitying. | 2.15 | 2.0 | 1.30 | .82 | -.50 | .25 | .54 |
| 17 |  | I am unable to get or keep a regular job. | 1.87 | 1.0 | 1.28 | 1.40 | .78 | .19 | .41 |
| 18 |  | I am emotionally unstable. | 2.48 | 2.0 | 1.30 | .47 | -.90 | .31 | .50 |
| 19 |  | I will never get away from alcohol. | 1.88 | 2.0 | 1.12 | 1.32 | 1.09 | .19 | .39 |
| 20 |  | I am unpredictable. | 1.72 | 1.0 | 1.11 | 1.43 | 1.09 | .15 | .37 |

**Table S4** Linear regression models included age^1^

|  | *Criterion variables* | | | | | | | | | | | | | | | | | |
| --- | --- | --- | --- | --- | --- | --- | --- | --- | --- | --- | --- | --- | --- | --- | --- | --- | --- | --- |
|  | Self-esteem (RSES) | | | | | | Shame | | | | | | Drinking-refusal self efficacy (KAZ-35) | | | | | |
|  | Model 1 | | Model 2 | | Model 3 | | Model 1 | | Model 2 | | Model 3 | | Model 1 | | Model 2 | | Model 3 | |
| n | 148 | | 147 | | 147 | | 154 | | 147 | | 147 | | 150 | | 144 | | 144 | |
| R² | .239 | | .375 | | .383 | | .259 | | .293 | | .302 | | .119 | | .176 | | .137 | |
| *Predictor variables* | Beta | p | Beta | p | Beta | p | Beta | p | Beta | p | Beta | p | Beta | p | Beta | p | Beta | p |
| Age | .13 | .082 | .05 | .500 | .10 | .133 | -.01 | .969 | -.01 | .944 | -.02 | .734 | .23 | .004 | .16 | .052 | .20 | .014 |
| Gender | .01 | .942 | .01 | .871 | .05 | .485 | -.22 | .003 | -.23 | .002 | -.24 | .001 | .07 | .398 | .07 | .350 | .09 | .289 |
| Depression (PHQ-8) | -.46 | <.001 | -.31 | <.001 | -.30 | <.001 | .44 | <.001 | .38 | <.001 | .37 | <.001 | -.22 | .005 | -.12 | .159 | -.16 | .062 |
| SSAD-SF apply |  |  | -.41 | <.001 |  |  |  |  | .16 | .048 |  |  |  |  | -.28 | .002 |  |  |
| SSAD-SF harm |  |  |  |  | -.41 | <.001 |  |  |  |  | .19 | .018 |  |  |  |  | -.17 | .058 |

^1^16 missing values in age replaced by mean imputation; SSAD-SF: Self-Stigma in Alcohol Dependence Scale short form; apply: self-application of stereotypes; harm: harmful consequences for self-esteem**Table S5** Linear regression models of subgroup analysis with abstinent cases^1^ included age^2^

|  | *Criterion variables* | | | | | | | | | | | | | | | | | |
| --- | --- | --- | --- | --- | --- | --- | --- | --- | --- | --- | --- | --- | --- | --- | --- | --- | --- | --- |
|  | Self-esteem (RSES) | | | | | | Shame | | | | | | Drinking-refusal self efficacy (KAZ-35) | | | | | |
|  | Model 1 | | Model 2 | | Model 3 | | Model 1 | | Model 2 | | Model 3 | | Model 1 | | Model 2 | | Model 3 | |
| n | 32 | | 31 | | 31 | | 32 | | 31 | | 31 | | 31 | | 30 | | 30 | |
| R² | .361 | | .443 | | .410 | | .197 | | .234 | | .198 | | .233 | | .226 | | .229 | |
| *Predictor variables* | Beta | p | Beta | p | Beta | p | Beta | p | Beta | p | Beta | p | Beta | p | Beta | p | Beta | p |
| Age | .21 | .194 | .13 | .436 | .16 | .357 | .11 | .556 | .04 | .845 | .11 | .561 | -.12 | .514 | -.11 | .593 | -.15 | .436 |
| Gender | -.10 | .551 | -.04 | .778 | .03 | .866 | -.13 | .460 | -.13 | .460 | -.17 | .400 | .42 | .029 | .39 | .047 | .44 | .042 |
| Depression (PHQ-8) | -.53 | .002 | -.31 | .103 | -.41 | .029 | .43 | .020 | .56 | .016 | .40 | .062 | -.22 | .220 | -.28 | .209 | -.19 | .359 |
| SSAD-SF apply |  |  | -.38 | .063 |  |  |  |  | -.26 | .269 |  |  |  |  | .08 | .724 |  |  |
| SSAD-SF harm |  |  |  |  | -.27 | .156 |  |  |  |  | .05 | .840 |  |  |  |  | -.11 | .630 |

^1^ self-reported abstinent phase over six months; ^2^16 missing values in age replaced by mean imputation; SSAD-SF: Self-Stigma in Alcohol Dependence Scale short form; apply: self-application of stereotypes; harm: harmful consequences for self-esteem
