# Supplementary material for: Self-stigma in alcohol dependence scale: development and validity of the short form
Source: BMC Psychiatry. 2024 Oct 25;24:735. doi: 10.1186/s12888-024-06187-z (PMC11515145; doi:10.1186/s12888-024-06187-z)
Supplement: Supplementary file 2 — Supplementary Material 2 [file 12888_2024_6187_MOESM2_ESM.docx]

| **Self-stigma in alcohol dependence** |
| --- |

There are many attitudes about people with alcohol problems. We would like to know what you think most of the public as a whole (or most people) believe about these attitudes. What do **most other people** think about someone with alcohol problems?

Please answer the following questions by ticking the appropriate box on the scale. 1 means: „I strongly disagree“, 5 means „I strongly agree“. You can grade your answer using any of the numbers between 1 and 5.

**Important:** This is about what you think **most other people** believe, regardless whether you personally share their opinion or not.

**Example:**

| **I think the public believes...** | **I strongly disagree**  **1** | **2** | **3** | **4** | **I strongly agree**  **5** |
| --- | --- | --- | --- | --- | --- |
| ... most people with alcohol problems are dangerous. | ① | ② | ③ | ④ | ⑤ |

If you strongly agree that most people think persons with alcohol problems are dangerous, you tick “5”.

| **I think the public believes...** | **I strongly disagree**  **1** | **2** | **3** | **4** | **I strongly agree**  **5** |
| --- | --- | --- | --- | --- | --- |
| 1. most people with alcohol problems   are unable to get or keep a regular  job. | ① | ② | ③ | ④ | ⑤ |
| 1. most people with alcohol problems are unpredictable. | ① | ② | ③ | ④ | ⑤ |
| 1. most people with alcohol problems   are emotionally unstable. | ① | ② | ③ | ④ | ⑤ |
| 1. most people with alcohol problems   will never get away from alcohol. | ① | ② | ③ | ④ | ⑤ |
| 1. most people with alcohol problems   are self-pitying. | ① | ② | ③ | ④ | ⑤ |

ssadsf101-105

© Georg Schomerus, Leipzig University, Germany

Now we would like to know how **you** currently feel about these attitudes. Do you

agree with them?

| **I think...** | **I strongly disagree**  **1** | **2** | **3** | **4** | **I strongly agree**  **5** |
| --- | --- | --- | --- | --- | --- |
| 1. most people with alcohol problems are unpredictable. | ① | ② | ③ | ④ | ⑤ |
| 1. most people with alcohol problems   will never get away from alcohol. | ① | ② | ③ | ④ | ⑤ |
| 1. most people with alcohol problems   are unable to get or keep a regular  job. | ① | ② | ③ | ④ | ⑤ |
| 1. most people with alcohol problems   are self-pitying. | ① | ② | ③ | ④ | ⑤ |
| 1. most people with alcohol problems   are emotionally unstable. | ① | ② | ③ | ④ | ⑤ |

ssadsf201-205

Next we would like to know if you currently think any of these attitudes **are true**

**about you**.

| **Because I have alcohol problems...** | **I strongly disagree**  **1** | **2** | **3** | **4** | **I strongly agree**  **5** |
| --- | --- | --- | --- | --- | --- |
| 1. I am emotionally unstable. | ① | ② | ③ | ④ | ⑤ |
| 1. I am unable to get or keep a   regular job. | ① | ② | ③ | ④ | ⑤ |
| 1. I am self-pitying. | ① | ② | ③ | ④ | ⑤ |
| 1. I will never get away from   alcohol. | ① | ② | ③ | ④ | ⑤ |
| 1. I am unpredictable. | ① | ② | ③ | ④ | ⑤ |

ssadsf301-305

© Georg Schomerus, Leipzig University, Germany

Finally, we would like to know how these attitudes currently affect your self-esteem or

**how much you respect yourself**.

| **I currently respect myself less, because…** | **I strongly disagree**  **1** | **2** | **3** | **4** | **I strongly agree**  **5** |
| --- | --- | --- | --- | --- | --- |
| 1. I am self-pitying. | ① | ② | ③ | ④ | ⑤ |
| 1. I am unable to get or keep a   regular job. | ① | ② | ③ | ④ | ⑤ |
| 1. I am emotionally unstable. | ① | ② | ③ | ④ | ⑤ |
| 1. I will never get away from   alcohol. | ① | ② | ③ | ④ | ⑤ |
| 1. I am unpredictable. | ① | ② | ③ | ④ | ⑤ |

ssadsf401-405

© Georg Schomerus, Leipzig University, Germany

**Self-Stigma Scale in Alcohol Dependence Short Form (SSAD-SF) Evaluation Instructions**

The SSAD-SF consists of 4 subscales built on each other and are intended to represent the process leading up to self-stigmatization:

1. Awareness being aware of associated stereotypes in public
2. Agreement agreeing with these stereotypes
3. Application applying the stereotypes to oneself
4. Resulting harm suffering lower self-esteem

Each subscale consists of 5 recurring stereotypes preceded by different introductory phrases. Subscale scores are summed up and ranging from 5 to 25 points.

Subscales 1. *aware* and 2. *agree* can be answered by people who do not identify as having a drinking problem and can be used in healthy populations. Subscales 3. *apply* and 4. *harm* are designed to measure the degree of self-sigma and can also be used separately for this purpose.

The subscales are intended to capture the different steps of stigma, therefore a calculation of the subscale scores is more meaningful and recommended. However, if an overall score is to be calculated, the 4 subscale scores are to be added up and divided by 4, so that a total value between 5 and 25 points is obtained.

For use in commercial and/or non-research purposes, please contact Prof. Dr. Georg Schomerus at: Georg.Schomerus@medizin.uni-leipzig.de
